# Relationships between Exercise Modality and Activity Restriction, Quality of Life, and Hematopoietic Profile in Korean Breast Cancer Survivors

**DOI:** 10.3390/ijerph17186899

**Published:** 2020-09-21

**Authors:** MunHee Kim, Wi-Young So, Jiyoun Kim

**Affiliations:** 1 Department of Health Science, Korea National Sport University, Seoul 05541, Korea; kmoonhee@hanmail.net; 2 Sports and Health Care Major, College of Humanities and Arts, Korea National University of Transportation, Chungju-si 27469, Korea; wowso@ut.ac.kr; 3 Department of Exercise Rehabilitation and Welfare, Gachon University, Incheon 21936, Korea

**Keywords:** aerobic exercise, obesity, resistance exercise, subjective health status, walking

## Abstract

This study aimed to examine the relationships between activity restriction, quality of life (QoL), and hematopoietic profile in breast cancer survivors according to exercise modality. The subjects in this study were 187 female breast cancer survivors among a total of 32,631 participants in the Korea National Health and Nutrition Examination Survey, which was conducted from 2016 to 2018. The selected subjects participated in a questionnaire survey and blood analysis. A cross-analysis was conducted to determine the relationship between participation in various modality of exercise (e.g., aerobic exercise, resistance exercise, walking exercise). The phi coefficients or Cramer’s V value for activity restriction and QoL were calculated; an independent *t*-test was conducted to evaluate the differences between hematopoietic profiles based on the modality of exercise. Statistically significant correlations were seen between obesity and aerobic exercise and walking frequency, as well as between diabetes and aerobic exercise and activity restriction. With respect to QoL, there was a statistically significant correlation between participation in aerobic exercise and exercise ability, participation in aerobic exercise and anxiety/depression, participation in resistance exercise and subjective health status, participation in resistance exercise and exercise ability, and participation in weekly walking exercise and self-care ability. Regarding hemodynamic changes, red blood cells increased significantly in breast cancer survivors who participated in weekly resistance exercise compared to in those who did not. In conclusion, exercise participation had a positive effect on activity restriction, QoL, and hematopoietic profile in breast cancer survivors; in particular, some modalities of aerobic exercise were more effective.

## 1. Introduction

Breast cancer is the most common cancer among Korean women. According to a report from the Korea Central Cancer Registry under the Ministry of Health and Welfare, the age-adjusted cancer incidence rate in 2016 was 62.6 out of 100,000 women, which is significantly higher than 54.7 in 2014 and 56.1 in 2015. When noted according to age group, breast cancer is most common among women in their 40s (44.3%), followed by those in their 50s (30.2%) and 60s (16.1%) [1]. The Korea Ministry of Health and Welfare reported that, while the number of patients with cancer who survived for more than 5 years after cancer diagnosis exceeded 1 million in 2017, and the cancer survival rate reached 70%, 40% of patients with breast cancer developed depression [1,2]. Breast cancer survivors experience many activity restrictions because of sexual problems, infertility, fatigue, appearance, separation or divorce from their spouses, fear of cancer recurrence and death, etc. [2,3,4,5,6].

Because of these psychological stresses, those with breast cancer tend not to actively participate in many activities, which leads to the deterioration of cardiovascular health, muscle strength, and bone health, thus increasing the risk of osteoporosis and cardiovascular disease [7]. These diseases eventually cause activity restrictions for breast cancer survivors, including discomfort in daily life and absenteeism due to concurrent diseases [8]. In fact, 48.4% of breast cancer survivors in Korea are obese, which increases the risk of breast cancer recurrence and mortality to 35–40%, and may cause insulin resistance, metabolic syndrome, and type 2 diabetes [9]. Since obesity and diabetes also increase the risk of other cancers, more care is required [10]. In recent studies, the expression and activity of iron-related proteins (ferritin, hepcidin, and ferroportin) in breast cancer cells affected the prognosis of breast cancer [11]. In particular, poor iron metabolism (anemia) in patients with breast cancer is a common phenomenon based on tumor stage and anticancer treatment used, and about 43–47% of patients with breast cancer develop anemia [12,13]. In addition, patients with cancer experience inflammatory reactions in their bodies due to obesity, as their level of activity decreases because of fatigue [14,15].

The prevention of cancer is of primary importance; however, women who already have cancer need proper physical and emotional care to maintain their quality of life (QoL). For cancer survivors, the ongoing management of lifestyle (nutrition, physical activity, sleep, stress) is important. Among lifestyle factors, physical activity is widely recognized as an effective non-pharmacological treatment for patients with cancer [16,17,18]. In order to manage or prevent breast cancer, various modalities of exercise are used. Therefore, this study, utilizing the Korea National Health and Nutrition Examination Survey (KNHANES) conducted from 2016 to 2018, aimed to examine the relationships between participation in various modalities of exercise and activity restriction, QoL, and hemodynamic changes in breast cancer survivors, and to determine which modality of exercise is more effective for breast cancer survivors.

## 2. Materials and Methods

### 2.1. Study Design and Participants

The KNHANES is a national survey conducted by trained experts every year under the supervision of the Korea Centers for Disease Control and Prevention. This study was conducted with 187 live female patients with breast cancer of 32,631 participants who participated in the KNHANES for 3 years. The mean age of the subjects and mean age at diagnosis were post-menopausal. For more information about the physical characteristics of the subjects, please see Table 1.

### 2.2. Physical Activity Assessment

The KNHANES physical activity levels were measured using 3 exercise categories: (1) aerobic exercise only (medium intensity aerobic activity of 150 min per week or high-intensity aerobic activity for 75 min per week); (2) resistance exercise only (>1 time per week); (3) walking exercise only (1–2/times per week, 3–5/times per week, 6–7/times per week). Each participant could belong to multiple exercise categories.

### 2.3. Activity Restriction

The questions asked during the health interview survey regarding activity restrictions consisted of: the presence of activity restriction, causes of activity restriction, diseases, and experience of absenteeism. To ascertain activity restriction, 5 questions (discomfort in the last 2 weeks, disease in the past month, disease in the last year, absenteeism in the last month, absenteeism in the last year) were asked, and were designed to be answered with a “yes” or ”no.” In addition, the causes of activity restriction, the presence of obesity, diabetes, and anemia (dizziness), which are closely associated with breast cancer, were examined.

### 2.4. Subjective Health Status and QoL

For subjective health status, the question “How do you think your health is in normal times?” was asked, and answers were scored from 1 point for “Very Bad” to 5 points for “Very Good” using a 5-point Likert scale. The higher the score, the better the perceived health status by the subject. The EuroQoL-5 dimension (EQ-5D) developed by the EuroQoL Group was used to measure QoL related to overall health. It consists of 5 multiple-choice questions concerning exercise ability, self-care, daily activities, pain/discomfort, and anxiety/depression. Each of the 5 questions can be answered with 1 of 3 responses: “No problem at all”, “There are some problems”, and “There are serious problems”. In this study, Cronbach’s alpha for the instrument was 0.78.

### 2.5. Blood Analysis

Fasting blood samples from all participants in the Korea National Health and Nutrition Examination Survey were collected; white blood cells, red blood cells (RBCs), hemoglobin, platelets, hematocrit, and hs-C-reactive protein (CRP) levels were analyzed. For diabetes, stage 3 diabetes (normal, impaired glucose tolerance (IGT) and diabetes) was based on blood glucose after fasting for more than 8 h. Anemia was determined based on a hemoglobin level <12 g/dL.

### 2.6. Ethics Statement

The KNHANES was conducted as an interview survey in which the investigators interviewed the subjects and collect responses to the questions. In this study, the raw data from the seventh survey (2016–2018) that met the criteria of the study were downloaded from the KNHANES website (http://knhanes.cdc.go.kr/). In order to use the data, protocols for using the raw data from the KNHANES website were followed. Since the KNHANES is considered a public welfare study conducted by the Korean government, this study was conducted without the prior approval of the Research Ethics Review Committee.

### 2.7. Statistical Analysis

Phi coefficients or Cramer’s V value were calculated using cross-analysis to determine whether there was a relationship between exercise participation, activity restriction, subjective health status, and QoL by exercise modality. An independent *t*-test was conducted to examine the differences between exercise modality participation by hematopoietic profile, and alpha (α) was set to 0.05. The reasons for the different case numbers for each variable is due to missing data from those who did not respond to the questionnaire. All analyses were conducted using SPSS version 18.0 (IBM Corp., Armonk, NY, USA).

## 3. Results

This study examined the relationships between exercise modality, activity restriction, subjective health status, QoL, and hematopoietic profile in breast cancer survivors who participated in the 2016–2018 KNHANES. The results of the cross-analysis, conducted to determine the correlation between exercise participation and activity restriction-related variants by exercise modality (aerobic exercise, resistance exercise, walking exercise) in the breast cancer survivors, are presented in Table 2. There were no statistically significant correlations between participation in various modalities of exercise and activity restriction (discomfort in the past 2 weeks, disease in the last month, disease in the last year, absenteeism in the last month, absenteeism in the last year) in the breast cancer survivors.

Among activity restriction due to disease, there was a statistically significant correlation between obesity and aerobic exercise participation (*p* < 0.046) and walking exercise frequency (*p* < 0.029). However, there was an exception; one subject who participated in aerobic and resistance exercises had a higher obesity rate than those who did not participate. There was also a significant correlation between diabetes and aerobic exercise participation at the level of *p* < 0.038. The subjects who participated in aerobic exercise showed a lower prevalence of diabetes compared to those who did not participate in aerobic exercise.

The results of the cross analysis, conducted to examine the correlation between subjective health status and QoL by exercise modality (aerobic exercise, resistance exercise, walking exercise) in breast cancer survivors, are shown in Table 3. There was a statistically significant correlation between subjective health status and resistance exercise participation at the level of *p* < 0.180. There was a statistically significant correlation between mobility, aerobic exercise participation, and resistance exercise participation at the levels of *p* < 0.028 and *p* < 0.026. There were also a statistically significant correlation between self-care and walking exercise frequency, and anxiety/depression and aerobic exercise participation at the levels of *p* < 0.037 and *p* < 0.017.

The independent *t*-test conducted to examine the effect of exercise participation on the hematopoietic profile by exercise modality in breast cancer survivors is presented in Table 4. The RBC was significantly higher at the level of *p* < 0.028 for those who participated in resistance exercise compared to those who did not.

## 4. Discussion

Breast cancer is affected by genetic and environmental factors, such as menarche, menopause, childbirth, and lactation experience; it is reported that a Western diet and inactive lifestyle increase the incidence [19]. Women who have undergone surgery because of the development of breast cancer, ovarian cancer, and uterine cancer may develop depression because they feel deprived of femininity, which may lead to family or social problems. In addition, it has been reported that the risk of myopathy, osteoporosis, and cardiovascular disease increases in breast cancer survivors [7].

Cancer survivors are recommended to participate in various modalities of exercise to prevent daily fatigue and cancer recurrence. In this study, there was no significant correlation between exercise participation and activity restriction-related discomfort or disease, and absenteeism for the last 2 weeks; however, there was a correlation between obesity and diabetes and activity restriction. Specifically, obesity and diabetes were significantly correlated with aerobic exercise participation and walking exercise frequency in breast cancer survivors. In this study, aerobic exercise and walking exercise showed a significantly positive correlation. It is suggested that aerobic exercise and walking (6–8 repetitions per week) are good solutions for obesity in breast cancer survivors. In addition, diabetes showed a correlation with aerobic exercise, showing that participation in aerobic exercise has a lower prevalence of diabetes compared with no participation in aerobic exercise. A meta-analysis conducted by Protani et al. [20], reported that the risk of cancer recurrence or death was 30% higher in breast cancer survivors who were obese than in breast cancer survivors of normal weight. It has also been reported that excess fat tissue caused by obesity increases the recurrence rate of breast cancer [21], and aerobic exercise (walking exercise) reduces the size of fat cells [22] and improves immune function [23]. However, increased fatigue due to a rapid increase in the level of activity may lower the immunity in patients with cancer, so care must be taken during exercise [24]. In addition, patients with breast cancer tend to lose muscle strength because of changes in body composition during anticancer treatment; resistance exercise has a positive effect on maintaining body composition and strength [25], indicating that breast cancer survivors need to participate in various modalities of exercise to further reduce cancer-related risk factors and prevent concurrent diseases.

In this study, there was a significant correlation between resistance exercise participation and subjective health status in breast cancer survivors. With respect to QoL, mobility and anxiety/depression were significantly correlated with aerobic exercise participation self-care, and walking exercise frequency. These results are similar to those of a study that reported a significant increase in QoL, fatigue, and depression symptoms after cancer survivors participated in exercise [26].

Regarding the examination of the relationship between exercise modality and the hematopoietic profile of the breast cancer survivors in this study, RBC significantly increased depending upon weekly resistance exercise participation; thus, those who participated in resistance exercise had higher RBC counts than those who did not. An increase in RBC count is closely associated with the prevalence of anemia. The blood cells of patients with cancer do not pass through blood vessels because of the deformation of red blood cells, which forms congestion and causes anemia. This phenomenon has been reported in more than 40–64% of patients with cancer [27,28]. Anemia can cause dizziness, weakness, and fatigue in everyday life, which may, in turn, deteriorate the QoL and restrict the activities of breast cancer survivors [29]. Although not statistically significant, it was found that the prevalence of anemia was higher in those who participated in all modality of exercise than in those who did not, as shown in Table 1, which is considered to be closely associated with increased RBCs, even though they were within the normal range. Mohamady et al. [30] and Drouin et al. [31] reported that participation in a 7-week exercise program prevented the increase in RBC and hemoglobin in patients with breast cancer who were undergoing radiation therapy. It has also been reported that exercise improves systemic inflammation in cancer survivors [32,33,34,35].

However, in this study, the inflammatory index hs-CRP was within the normal range for all exercise modality, and there was no difference. The results of this study found that participation in physical activities (aerobic exercise, resistance exercise, walking exercise) lowered the prevalence of obesity and diabetes affecting patients with breast cancer. Physical activity participation improved subjective health status and exercise ability, and reduced depression and anxiety, thus improving the quality of life of breast cancer survivors in Korea. Among the modalities of exercise assessed, aerobic exercise had a greater positive correlation, indicating that it may be more effective.

There are several limitations to this study. First, the amount of exercise participation was not directly measured by objective observance, but surveyed indirectly by using a questionnaire. Second, there was a lack of a methodological approach for measuring the proper amount of exercise according to the grade of breast cancer and cancer therapy method, suggesting the necessity of a follow-up study. Third, this study was conducted only with patients with breast cancer; thus, the findings cannot be generalized to other cancer patients. Fourth, additional physical activity evaluations, such as activities of daily living or instrumental activities of daily living, were not conducted, suggesting the necessity for a further study with additional variables. However, combining resistance exercise and aerobic exercise to lessen muscle weakening is recommended. The habit of performing exercise on a regular basis is considered most important for breast cancer survivors for the prevention of cancer recurrence and for cancer recovery.

## 5. Conclusions

This study found that, for breast cancer survivors, participation in physical activity, such as aerobic exercise, resistance exercise, and walking exercise may lower the prevalence of diseases such as obesity and diabetes. Furthermore, physical activity can reduce depression and anxiety and improve subjective health status, exercise ability, and quality of life. In particular, aerobic exercise was shown to be effective in positively affecting a number of variables, but resistance training is also recommended to prevent muscle loss. The effort to establish regular exercise habits, regardless of modality, seems to be important for the mental and physiological health of breast cancer survivors.

## Figures and Tables

**Table 1:** The characteristics of the study subjects.

Variables	Mean ± Standard Deviation
Breast cancer survivor (*n*)	187
Age (years)	60.8 ± 11.1
Breast cancer diagnosis age (years)	54.5 ± 11.3
Menarche age (years)	14.3 ± 1.8
Menopause age (years)	48.00 ± 4.9
Height (cm)	155.8 ± 6.1
Weight (kg)	57.5 ± 8.3

**Table 2:** Correlation between exercise modality and activity restriction-related variants in breast cancer survivors.

		Aerobic Exercise % (Frequency)		Resistance Exercise % (Frequency)		Walking % (Frequency)		
		Yes	No	Yes	No	1–2/Wk.	3–5/Wk.	6–7/Wk.
Discomfort in the last 2 weeks	Yes	10.8% (20)	18.3% (34)	4.81% (9)	24.5% (46)	12.6% (15)	10.9% (13)	9.2% (11)
	No	32.3% (60)	38.7% (72)	13.9% (26)	56.9% (106)	21.0% (25)	27.7% (33)	18.5% (22)
	Phi coefficients	0.077		0.039		0.084		
Presence of disease in the last month	Yes	5.9% (11)	4.3% (8)	2.7% (5)	8.0% (15)	5.0% (6)	5.9% (7)	2.5% (3)
	No	37.1% (69)	53.7% (98)	16.0% (30)	73.2% (137)	28.6% (34)	32.8% (39)	25.2% (30)
	Phi coefficients	0.101		0.056		0.079		
Presence of disease in the last year	Yes	13.1% (8)	16.4% (10)	3.2% (2)	24.2% (16)	15.8% (6)	13.2% (5)	5.3% (2)
	No	39.3% (24)	31.1% (19)	24.6% (15)	45.9% (28)	13.2% (5)	23.7% (9)	28.9% (11)
	Phi coefficients	0.104		0.242		0.130		
Presence of absenteeism in the last month	Yes	5.3% (4)	2.7% (2)	2.7% (2)	5.3% (4)	3.8% (2)	3.8% (2)	3.8% (2)
	No	36.0% (27)	56.0% (42)	13.3% (10)	78.7% (59)	32.1% (17)	29.3% (15)	28.3% (15)
	Phi coefficients	−0.152		−0.101		0.019		
Presence of absenteeism in the last year	Yes	0.0% (0)	11.1% (3)	0.0% (0)	16.7% (5)	15.0% (3)	0.0% (0)	0.0% (0)
	No	37.0% (10)	51.9% (14)	10.0% (3)	63.3% (19)	25.0% (5)	25.0% (5)	35.0% (7)
	Phi coefficients	0.356		0.210		0.437		
Obesity	Underweight	0.5% (1)	2.2% (4)	0.0% (0)	3.2% (3)	0.8% (1)	2.3% (4)	0.0% (0)
	Normal	25.8% (48)	29.5% (55)	8.4% (8)	44.2% (42)	16.8% (20)	22.7% (27)	16.8% (20)
	Pre-obesity	11.8% (22)	9.1% (17)	5.3% (5)	15.8% (15)	5.9% (7)	5.9% (7)	10.0% (12)
	Obesity	4.8% (9)	16.1% (30)	2.1% (7)	21.1% (20)	10.1% (12)	6.7% (8)	0.8% (1)
	Cramer’s V	0.246 *		0.158		0.290 *		
Presence of diabetes	Normal	25.5% (40)	36.3% (57)	14.5% (23)	47.7% (75)	18.4% (19)	30.1% (31)	22.3% (23)
	IGT	15.9% (25)	8.9% (14)	5.0% (8)	19.6% (31)	8.7% (9)	7.7% (8)	2.9% (3)
	Diabetes	5.1% (8)	8.3% (13)	0.6% (1)	12.7% (20)	2.9% (3)	3.9% (4)	2.9% (3)
	Cramer’s V.	0.204 *		0.177		0.487		
Presence of anemia	Yes	39.1% (63)	50.3% (81)	7.5% (28)	72.5% (116)	26.7% (28)	35.2% (37)	30.5% (32)
	No	6.2% (10)	4.3% (7)	1.8% (3)	8.7% (14)	2.9% (3)	4.7% (5)	0.0% (0)
	Phi coefficients	0.093		0.159		0.141		

IGT: impaired glucose tolerance; * *p* < 0.05; cross-analysis.

**Table 3:** Correlation between exercise modality, subjective health status and QoL in breast cancer survivors.

			Aerobic Exercise % (Frequency)		Resistance Exercise % (Frequency)		Walking % (Frequency)		
			Yes	No	Yes	No	1–2/Wk.	3–5/Wk.	6–7/Wk.
Subjective health status		Very Good	2.7% (5)	1.1% (2)	1.1% (2)	2.7% (5)	0.0% (0)	0.0% (0)	0.0% (0)
		Good	7.0% (13)	7.0% (15)	1.6% (3)	13.4% (25)	4.2% (5)	8.4% (10)	3.4% (4)
		Normal	25.3% (47)	28.5% (53)	14.4% (27)	39.0% (73)	16.8% (20)	18.5% (22)	15.7% (19)
		Bad	5.4% (10)	12.4% (23)	1.1% (2)	16.6% (31)	6.7% (6)	8.4% (10)	5.9% (7)
		Very bad	2.7% (5)	7.0% (13)	0.5% (1)	9.6% (18)	7.6% (9)	3.4% (4)	2.5% (3)
		Cramer’s V	0.194		0.252 *		0.401		
QoL	Mobility	No difficulty walking	37.0% (69)	40.3% (75)	17.6% (33)	59.4% (111)	22.7% (27)	31.0% (37)	21.8% (26)
		Walking is a bit difficult	5.9% (11)	15.1% (28)	1.1% (2)	19.8% (37)	7.6% (9)	7.6% (9)	5.8% (7)
		Lying all day	0.0% (0)	1.6% (3)	0.0% (0)	2.1% (4)	3.4% (4)	0.0% (0)	0.0% (0)
		Cramer’s V	0.196 *		0.198 *		0.189		
	Self-care	No problems taking a bath/dressing	53.2% (99)	42.5% (79)	18.7% (35)	76.5% (143)	28.6% (34)	37.8% (45)	26.9% (32)
		Taking a bath/dressing somewhat hindered	3.2% (8)	0.5% (1)	0.0% (0)	4.3% (8)	5.0% (6)	0.8% (1)	0.8% (1)
		Not able to take a bath/dress alone	0.5% (1)	0.0% (0)	0.0% (0)	0.5% (1)	0.0% (0)	0.0% (0)	0.0% (0)
		Cramer’s V	0.197		0.108		0.037 *		
	Usual activities	No ADL disruption	38.1% (71)	48.9% (91)	17.6% (33)	68.9% (129)	25.2% (30)	35.3% (42)	24.4% (29)
		ADL is somewhat impeded	4.8% (9)	8.0% (15)	1.0% (2)	12.3% (23)	8.4% (10)	3.3% (4)	3.3% (4)
		Phi coefficients	−0.043		−0.108		0.093		
	Pain/ discomfort	No pain/ discomfort	27.9% (52)	37.6% (70)	13.9% (26)	51.3% (96)	18.5% (22)	27.7% (33)	17.6% (21)
		Some pain/ discomfort	13.4% (25)	16.3% (30)	4.3% (8)	25.7% (48)	10.9% (13)	10.1% (12)	7.5% (9)
		Severe pain/ discomfort	1.6% (3)	3.2% (6)	0.5% (1)	4.3% (8)	4.2% (5)	0.8% (1)	2.5% (3)
		Cramer’s V	0.785		0.093		0.136		
	Anxiety/ depression	No anxiety/depression	39.2% (73)	45.1% (84)	66.3% (33)	66.3% (124)	26.0% (31)	34.4% (41)	23.5% (28)
		Some anxiety/ depression	2.1% (4)	10.7% (20)	1.1% (2)	12.3% (23)	5.9% (7)	4.2% (5)	2.5% (3)
		Severe anxiety/ depression	1.6% (3)	1.0% (2)	0.0% (0)	2.7% (5)	1.7% (2)	0.0% (0)	1.7% (2)
		Cramer’s V	0.209 *		0.138		0.132		

* *p* < 0.05, tested by cross-analysis.

**Table 4:** Differences between exercise modality participation in relation to hemodynamic variables in breast cancer survivors.

Variables	Aerobic Exercise % (Frequency)		Resistance Exercise % (Frequency)		Walking % (Frequency)		
	Yes	No	Yes	No	1–2/Wk.	3–5/Wk.	6–7/Wk.
White blood cell (Thous/uL)	5.51 ± 1.30	5.57 ± 1.65	5.42 ± 1.71	5.54 ± 1.49	5.86 ± 1.92	5.12 ± 1.35	5.60 ± 1.22
Red blood cell (Mil/uL)	4.27 ± 0.40	4.29 ± 0.33	4.41 ± 0.38 *	4.25 ± 0.35	4.30 ± 0.37	4.33 ± 0.36	4.40 ± 0.38
Hemoglobin (g/dL)	12.97 ± 0.97	13.19 ± 0.87	12.81 ± 2.50	13.05 ± 0.91	13.1 ± 1.00	13.0 ± 0.93	13.35 ± 0.90
Platelets (Thous/uL)	246.34 ± 64.85	233.15 ± 62.36	242.65 ± 71.42	237.75 ± 64.73	220.45 ± 53.27	240.45 ± 65.52	252.00 ± 73.49
Hematocrit (%)	39.70 ± 3.00	40.36 ± 2.77	39.94 ± 5.48	39.86 ± 2.84	40.08 ± 3.06	40.09 ± 2.95	40.84 ± 3.03
C-reactive protein (mg/L)	1.55 ± 2.75	1.14 ± 3.01	1.18 ± 2.07	0.73 ± 0.30	0.93 ± 0.92	0.78 ± 0.86	1.42 ± 3.71

* *p* < 0.05; tested by an independent *t*-test.
